# Bicuspidization using autologous pericardium for neonatal quadricuspid truncal valve

**DOI:** 10.1186/s44215-023-00075-w

**Published:** 2023-10-09

**Authors:** So Motono, Akihiko Higashida, Masaya Aoki, Naoki Yoshimura

**Affiliations:** Department of Pediatric Cardiovascular Surgery, Toyama University Hospital, 2630 Sugitani, Toyama, 930-0194 Japan

**Keywords:** Quadricuspid truncal valve, Truncal valve insufficiency, Bicuspidization, Leaflet *reconstruction*

## Abstract

**Background:**

Neonatal truncal valve plasty remains a significant challenge. Several surgical techniques have been reported, but there is no standard procedure. We herein report a novel operative technique for the quadricuspid truncal valve with axisymmetric morphology.

**Case presentation:**

A 17-day-old neonate with severe truncal valve insufficiency developed persistent congestive heart failure and underwent urgent truncal valve plasty. The truncal valve was quadricuspid with four equal sinuses, and all four cusps were hypoplastic, resulting in a complete lack of coaptation. We performed bicuspidization valvuloplasty with leaflet *reconstruction* using autologous pericardium for this valve. The patient had an uneventful recovery and was free from recurrent truncal valve insufficiency for 10 months.

**Conclusions:**

Bicuspidization with leaflet *reconstruction* is a suitable surgical option for overcoming neonatal truncal valve insufficiency in cases with quadricuspid truncal valves with axisymmetric morphologies. Although the durability of a bicuspid neo-leaflet using glutaraldehyde-treated autologous pericardium is unknown, this technique is expected to postpone subsequent truncal valve replacement in such neonates.

## Background

Neonatal truncal valve plasty remains a significant challenge for congenital cardiovascular surgeons. In the patients with truncal valve insufficiency, the truncal valve can be dysplastic or dysfunctional or have an abnormal number of cusps, usually four. Several surgical techniques have been reported, but there is no standard procedure [1–4].

We herein report a successful case of truncal valve repair with bicuspidization using glutaraldehyde-treated autologous pericardium for the neonatal quadricuspid truncal valve.

## Case report

The patient was born at 40 weeks’ gestation with a birth weight of 3012 g. The postnatal diagnosis by transthoracic echocardiography was truncus arteriosus (Collet-Edwards II) with severe truncal valve insufficiency. The patient underwent bilateral pulmonary artery bandings at 3 days old. *Pericardium was incised on the right side, repaired by tight interrupted sutures, and covered by expanded polytetrafluoroethylene sheet for future use of truncal valve plasty.* However, severe truncal valve insufficiency complicated her postoperative course. The patient could not be weaned from mechanical ventilation, and tube feeding could not be increased due to severe congestive heart failure. We therefore decided to perform urgent truncal valve repair at 17 days old.

**Fig. 1:**
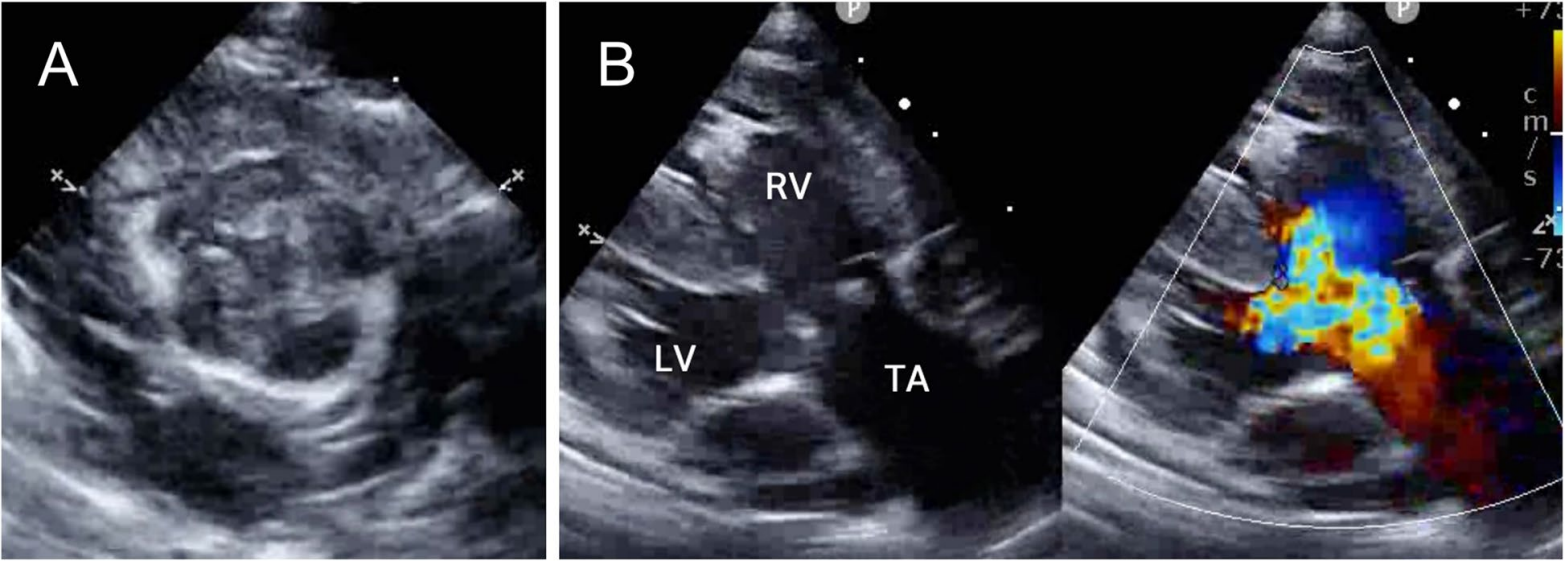
**A** Preoperative truncal valve in the parasternal short-axis view. **B** Color Doppler echocardiography shows significant truncal valve regurgitation. RV, right ventricle; LV, left ventricle; TA, truncus arteriosus

The operative technique used to repair the truncal valve was planned ahead of time with the help of two-dimensional echocardiography. The patient had a large quadricuspid truncal valve (15 mm in diameter) with 4 equal sinuses (type A Hurwitz and Roberts classification [5]). All four cusps were hypoplastic and the same size, resulting in a complete lack of a coaptation, which caused severe regurgitation (Fig. 1). The left and right coronary arteries arose from the right-posterior and left-anterior sinus, respectively. We performed bicuspidization using glutaraldehyde-treated autologous pericardium for this valve.

**Fig. 2:**
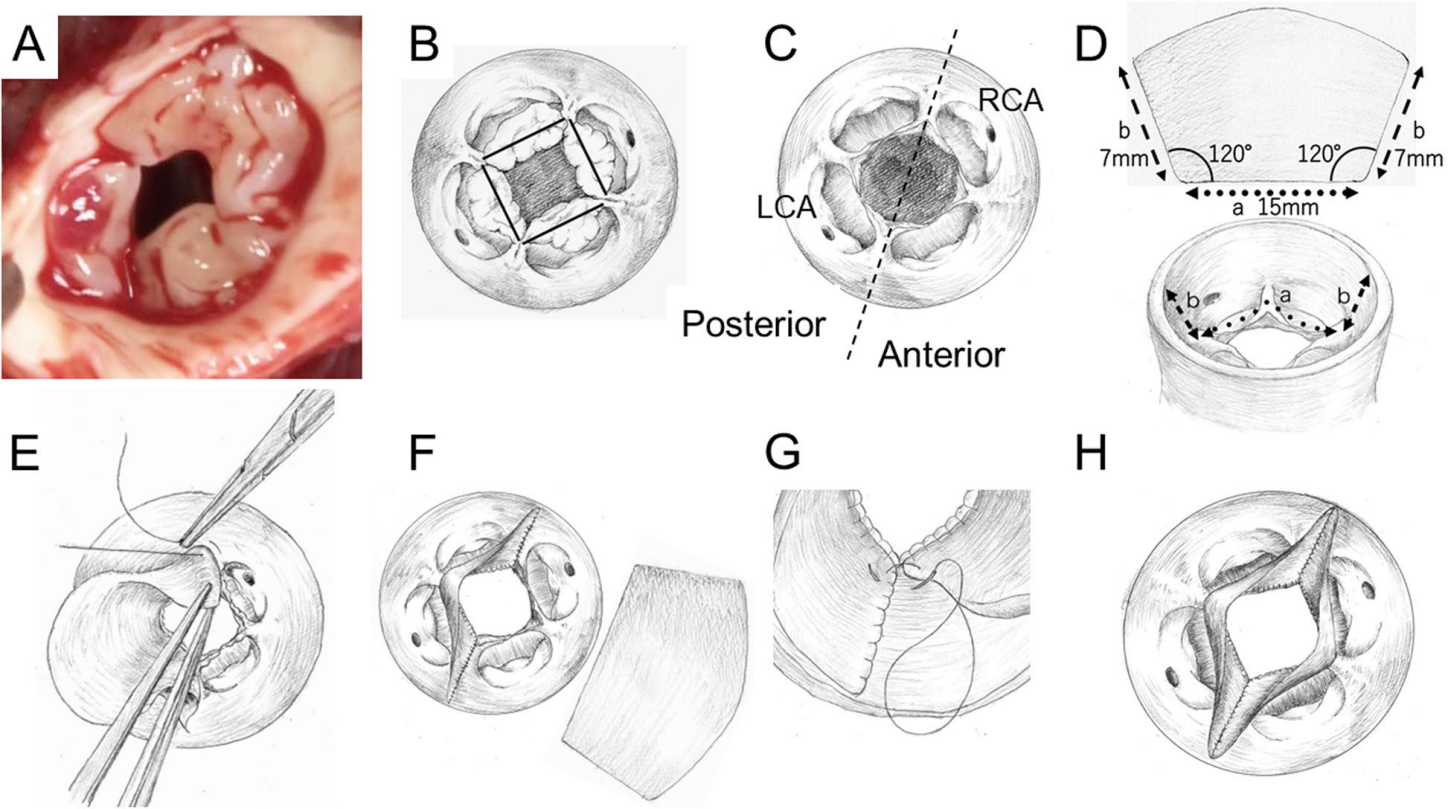
**A** In vivo picture of the quadricuspid truncal valve. **B** and **C** The diseased cusps are removed. **D** Neo-leaflets with a trapezoid-shaped base and a tapered fan-shaped top are tailored using autologous pericardium. The lengths of the base and side are determined based on the excised margin of two cusps (a) and the height from the sino-tubular junction to the stump of the truncal root (b). The angle from the base to the side is adjusted to approximately 120°. **E** The base of the posterior neo-leaflet is anastomosed continuously along the margin of the two posterior cusps. Both sides of the posterior neo-leaflet are sutured at the truncal wall from the commissures toward the stump of the truncal root. **F** The base of the anterior neo-leaflet is sutured at the two anterior cusps. **G** Both sides of the anterior neo-leaflet are secured by suturing both the aortic wall and posterior neo-leaflet to create the neo-commissures. **H** Schematic view of the reconstructed truncal valve

Through median sternotomy, a piece of pericardium was harvested, and the adventitia was aggressively removed, treated with buffered *0.6*% glutaraldehyde solution for 5 min, and then rinsed 3 times in saline for 5 min. Cardiopulmonary bypass with moderate hypothermia was established using distally placed aortic cannula and bicaval cannulas. Branch pulmonary arteries were mobilized as much as possible. The truncal root was cross-clamped, and transection of the truncal root was begun on the left side, 7 mm above the sino-tubular junction of the truncal valve. Cardioplegic arrest was obtained by selective coronary artery perfusion. The incision was extended carefully with direct visualization of both the coronary ostia and bilateral pulmonary orifices. Detachment of the ascending aorta, truncal root, and an ellipse of the truncal wall, including the bilateral pulmonary orifices, was completed. Intraoperative inspection of the truncal valve was confirmed via a preoperative evaluation, and the tips of all four cusps were significantly hypertrophied without commissural fusion (Fig. 2A). The diseased cusps were carefully removed (Fig. 2B, C). Two symmetrical neo-leaflets with a trapezoid-shaped base and a fan-shaped top were tailored using glutaraldehyde-treated autologous pericardium (Fig. 2D). The lengths of the base (15 mm) and side (7 mm) were determined by referencing the excised margin of 2 cusps and the height from the sino-tubular junction to the stump of the truncal root. The angle from the base to the side was adjusted to approximately 120°. Thereafter, the base of the neo-leaflet was anastomosed continuously along the margin of the two posterior cusps (Fig. 2E). Both sides of the neo-leaflet were sutured at the truncal wall from the right and left commissures toward the stump of the truncal root (Fig. 2F). For the other neo-leaflet, the base was anastomosed at the two anterior cusps. Specific focus was placed on the side anastomosis of the anterior neo-leaflet. Both sides of the anterior neo-leaflet were secured by suturing both the aortic wall and posterior neo-leaflet to create the neo-commissures (Fig. 2G, H). The ascending aorta and truncal root were anastomosed directly. Right ventricular-to-pulmonary artery reconstruction was performed using a hand-made mono-cuspid conduit with a 6-mm Gore-Tex graft (W.L. Gore and Associates, Inc., AZ, USA).

**Fig. 3:**
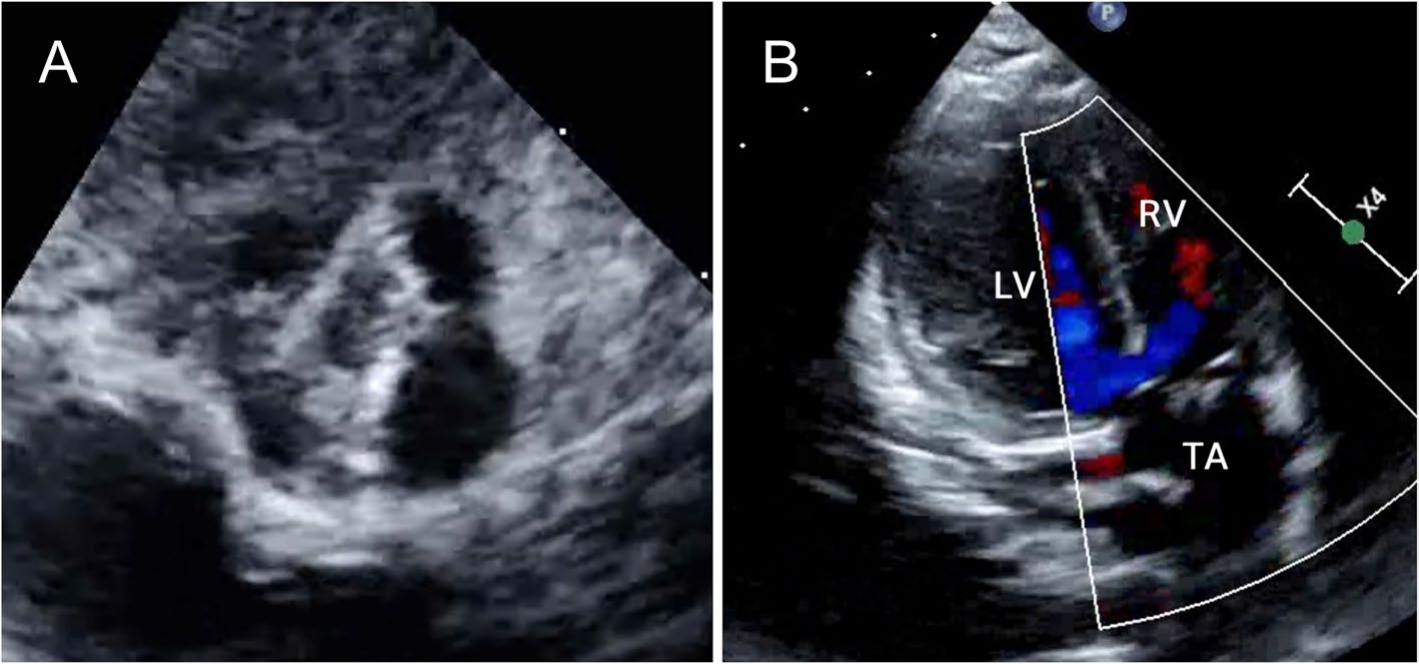
**A** The reconstructed truncal valve in the parasternal short-axis view. **B** Postoperative color-Doppler echocardiography shows complete elimination of the regurgitant jet of the truncal valve. RV, right ventricle; LV, left ventricle; TA, truncus arteriosus

Weaning from cardiopulmonary bypass was uneventful. The aortic cross-clamp time was 224 min, and the cardiopulmonary bypass time was 309 min. Intraoperative transesophageal echocardiography confirmed the elimination of truncal valve regurgitation without stenosis (Fig. 3). The patient had an uneventful recovery and was free from recurrent truncal valve insufficiency for 10 months.

However, truncal valve stenosis gradually progressed. Peak velocity through the truncal valve was 3.9 m/s on the latest echocardiography. The patient is awaiting a second truncal valve plasty.

## Discussion

Truncal valve surgery is inevitable in most patients with moderate or greater truncal valve insufficiency. Early surgical solutions include a mechanical valve or homograft replacement. However, repair of the truncal valve is clearly the better surgical strategy, especially for neonates.

Baird et al. described their experience of 57 patients [1]. They applied the tri-leaflet Ozaki procedure for congenital aortic and truncal valve reconstruction and reported excellent short-term results. However, the indication of the Ozaki technique on neonates remains controversial. Naimo, Fricke, and Lee reported the outcomes of 56 patients with quadricuspid truncal valves, including 8 neonates who underwent truncal valve surgery at the time of truncus arteriosus repair [2]. Of those eight neonates, three underwent tricuspidization with cusp resection and annulus reduction. Although their outcomes were also excellent, suitable anatomical variations for tricuspidization with cusp resection remain unclear. The detailed morphology of the truncal valve in these cases was not described. Boburg et al. advocated that cusp resection is recommended in patients with one hypoplastic sinus without coronary ostium [3].

To determine the appropriate operative technique, pre- and intraoperative evaluations of the morphology of the truncal valve are essential. In our case, leaflet *reconstruction* was inevitable, resection of a huge sinus with annular reduction was thought to carry a potential risk of coronary artery distortion and kinking, and dividing the truncal valve into two symmetrical regions was feasible. We therefore performed bicuspidization with leaflet *reconstruction*, considering it to be more beneficial than the other procedures [1–3].

The limitation of this study is that the technique described here does not imitate a true anatomical tri-leaflet semilunar valve. However, we believe that bicuspidization with leaflet *reconstruction* is a suitable surgical option for overcoming neonatal truncal valve insufficiency in cases with quadricuspid truncal valves with axisymmetric morphologies [4]. Although the durability of a bicuspid neo-leaflet using glutaraldehyde-treated autologous pericardium is unknown, this technique is expected to postpone subsequent truncal valve replacement in such neonates.

## Data Availability

Please contact the author for data request.
